# Underlying Data for Sequencing the Mitochondrial Genome with the Massively Parallel Sequencing Platform Ion Torrent^™^ PGM^™^

**DOI:** 10.1186/1471-2164-16-S1-S4

**Published:** 2015-01-15

**Authors:** Seung Bum Seo, Xiangpei Zeng, Jonathan L King, Bobby L Larue, Mourad Assidi, Mohamed H Al-Qahtani, Antti Sajantila, Bruce Budowle

**Affiliations:** 1 Institute of Applied Genetics, Department of Molecular and Medical Genetics, University of North Texas Health Science Center, 3500 Camp Bowie Blvd., Fort Worth, Texas, US 76107; 2 Center of Excellence in Genomic Medicine Research (CEGMR), King Abdulaziz University, Jeddah, Saudi Arabia; 3 KACST Technology Innovation Center for Personalized Medicine at King AbdulAziz University, Jeddah, Saudi Arabia; 4 Department of Forensic Medicine, Hjelt Institute, University of Helsinki, PO Box 40 (Kytösuontie 11), FI-00014 Helsinki, Finland

## Abstract

**Background:**

Massively parallel sequencing (MPS) technologies have the capacity to sequence targeted regions or whole genomes of multiple nucleic acid samples with high coverage by sequencing millions of DNA fragments simultaneously. Compared with Sanger sequencing, MPS also can reduce labor and cost on a per nucleotide basis and indeed on a per sample basis. In this study, whole genomes of human mitochondria (mtGenome) were sequenced on the Personal Genome Machine (PGM^TM^) (Life Technologies, San Francisco, CA), the out data were assessed, and the results were compared with data previously generated on the MiSeq^TM^ (Illumina, San Diego, CA). The objectives of this paper were to determine the feasibility, accuracy, and reliability of sequence data obtained from the PGM.

**Results:**

24 samples were multiplexed (in groups of six) and sequenced on the at least 10 megabase throughput 314 chip. The depth of coverage pattern was similar among all 24 samples; however the coverage across the genome varied. For strand bias, the average ratio of coverage between the forward and reverse strands at each nucleotide position indicated that two-thirds of the positions of the genome had ratios that were greater than 0.5. A few sites had more extreme strand bias. Another observation was that 156 positions had a false deletion rate greater than 0.15 in one or more individuals. There were 31-98 (SNP) mtGenome variants observed per sample for the 24 samples analyzed. The total 1237 (SNP) variants were concordant between the results from the PGM and MiSeq. The quality scores for haplogroup assignment for all 24 samples ranged between 88.8%-100%.

**Conclusions:**

In this study, mtDNA sequence data generated from the PGM were analyzed and the output evaluated. Depth of coverage variation and strand bias were identified but generally were infrequent and did not impact reliability of variant calls. Multiplexing of samples was demonstrated which can improve throughput and reduce cost per sample analyzed. Overall, the results of this study, based on orthogonal concordance testing and phylogenetic scrutiny, supported that whole mtGenome sequence data with high accuracy can be obtained using the PGM platform.

## Background

Forensic genetic analyses provide useful information on individuals that may or may not be associated with biological evidence found at crime scenes, identification of individuals who are missing or from mass disasters, and inferences related to cause and manner of death. Short tandem repeat (STR) loci, single nucleotide polymorphisms (SNPs) and lineage markers (primarily residing within the mitochondrial DNA (mtDNA) genome and Y chromosome) are the markers systems primarily used in forensic DNA typing and human identification [1-7]. The mtDNA genome (mtGenome) is a marker of choice for human identification, especially where forensic biologic evidence contains too little or no nuclear DNA, such as a hair shaft without root, a fingernail and old bones. Because of a lack of recombination in the mtGenome, this marker is particularly informative in kinship analyses where the maternal association being investigated may be separated by several generations. Sanger sequencing [8] and separation by capillary electrophoresis have been the standard method for mtDNA sequencing [9-11]. However, current mtDNA typing protocols are labor intensive, time consuming, and relatively costly. Therefore, most application-oriented laboratories tend to focus only on a portion of the mtGenome, i.e., the non-coding hypervariable regions. More discrimination power could be attained if more efficient and cost effective technologies allow expansion of genetic interrogation to the entire mtGenome.

Massively parallel sequencing (MPS) technology, also known as next generation sequencing, has become a viable and practical tool for biological research and application, such as in disease diagnosis [12], personalized medicine [13], species identification [14], evolutionary studies [15], and population studies [16]. MPS technologies have the capacity to sequence targeted regions or whole genomes of multiple nucleic acid samples with high coverage by sequencing millions of DNA fragments in a massively-parallel fashion. In fact, 2 to 96 different samples can be sequenced simultaneously using commercial barcoding kits, such as Ion Xpress Barcode kit (Life Technologies) and Nextera XT Index kit (Illumina). MPS platforms make possible higher throughput sequencing compared with Sanger sequencing at a substantially reduced cost on a per nucleotide basis and indeed on a per sample basis. In forensics, Parson et al. [17] demonstrated that sequence results with the Ion Torrent Personal Genome Machine (PGM™) (Ion Torrent, Life Technologies, San Francisco, CA) were highly concordant with those obtained with Sanger sequencing. Sanger sequencing is recognized as the gold standard for mtDNA sequencing and it would seem reasonable to compare new technologies with it for concordance testing. However, the gold standard status does not necessarily translate to a result (or in this context a base call) being correct. For example, Harismendy et al. [18] reported that Sanger sequencing generated 0.9% false negative and 3.1% false positive SNPs compared with three MPS platforms and one microarray platform. Moreover, the lower throughput of Sanger sequencing makes it impractical for concordance testing, and hence validation of whole mtGenome sequencing by MPS. Typically, only a small region of the mtDNA genome can be assessed by both approaches within a reasonable time and cost. Instead concordance testing of a MPS system may be achieved better by testing with an orthogonal MPS technology. King et al. [19] reported highly reliable whole mtGenome sequencing using long PCR, Nextera XT library preparation, and MPS with the MiSeq system (Illumina, San Diego, CA). A total of 283 mtDNA genomes were generated, the data were analyzed with multiple software tools, and the haplotype data were assessed phylogenetically. In addition, a subset of the samples were typed by Sanger sequencing at hypervariable regions (HV1 and HV2) and whole genome results from a cell line sample (data not shown) were compared with published literature; all base calls were concordant (excluding heteroplasmy). While the data reported by King et al. [19] are considered reliable, concordance testing of whole genome data would increase the confidence in MPS results. The PGM has been shown to provide quality mtDNA sequence results, and the results have been compared with Sanger sequencing generated data [17]. It now is feasible to perform orthogonal MPS concordance testing of whole mtDNA genome analyses in a high throughput, timely and cost-efficient fashion. Moreover, concordance testing permits evaluation and improvement of both systems. Results that are consistent between the two MPS systems can be considered reliable, and efforts can be focused on the differences to improve one, the other, or both systems. In this study, whole mtGenome sequencing was performed on the PGM to determine its feasibility, accuracy, and reliability. These results were compared with sequence data previously generated on the MiSeq [19], and the findings demonstrated that reliable base calling can be obtained by the PGM system as well.

## Results and discussion

In this study, 6 samples were multiplexed and sequenced at one time on a 314 chip (10 megabase throughput). The average throughout of 4 chips was 84 Mb (± 17), and the average total reads was 448,129 (± 78,773). Sufficient coverage was obtained to reliably determine the sequence for the entire mtGenome of six pooled libraries. In all, 24 samples were sequenced successfully on 4 chips. The number of samples that can potentially be sequenced simultaneously is determined by throughput and the lowest coverage region that allows for reliable variant calls at all sites.

### Coverage variation

**Figure 1:**
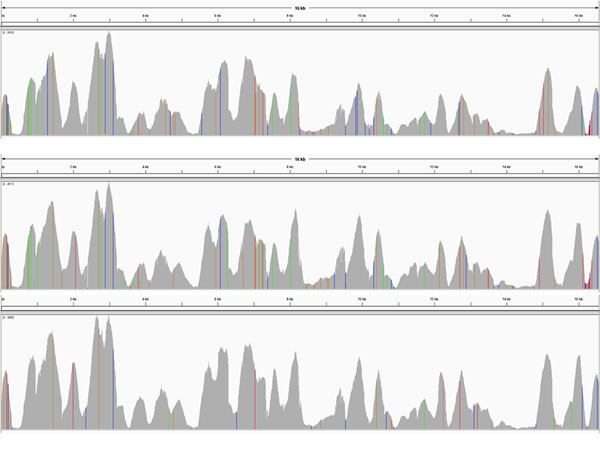
**A concentric Circos plot of the mtGenome.** A concentric Circos plot of the mtGenome representing mean coverage (outer circle; n=24); homopolymers, n≥4 bases, per region nucleotide position (middle circle; n=24); and mean coverage differentiated by reverse (dark) or forward (light) strand (inner circle; n=24). The rose diagram in the center is included for nucleotide position orientation and scale bars are included to the left of the individual plots to approximate values. The control region is offset slightly for orientation.

**Figure 2:**
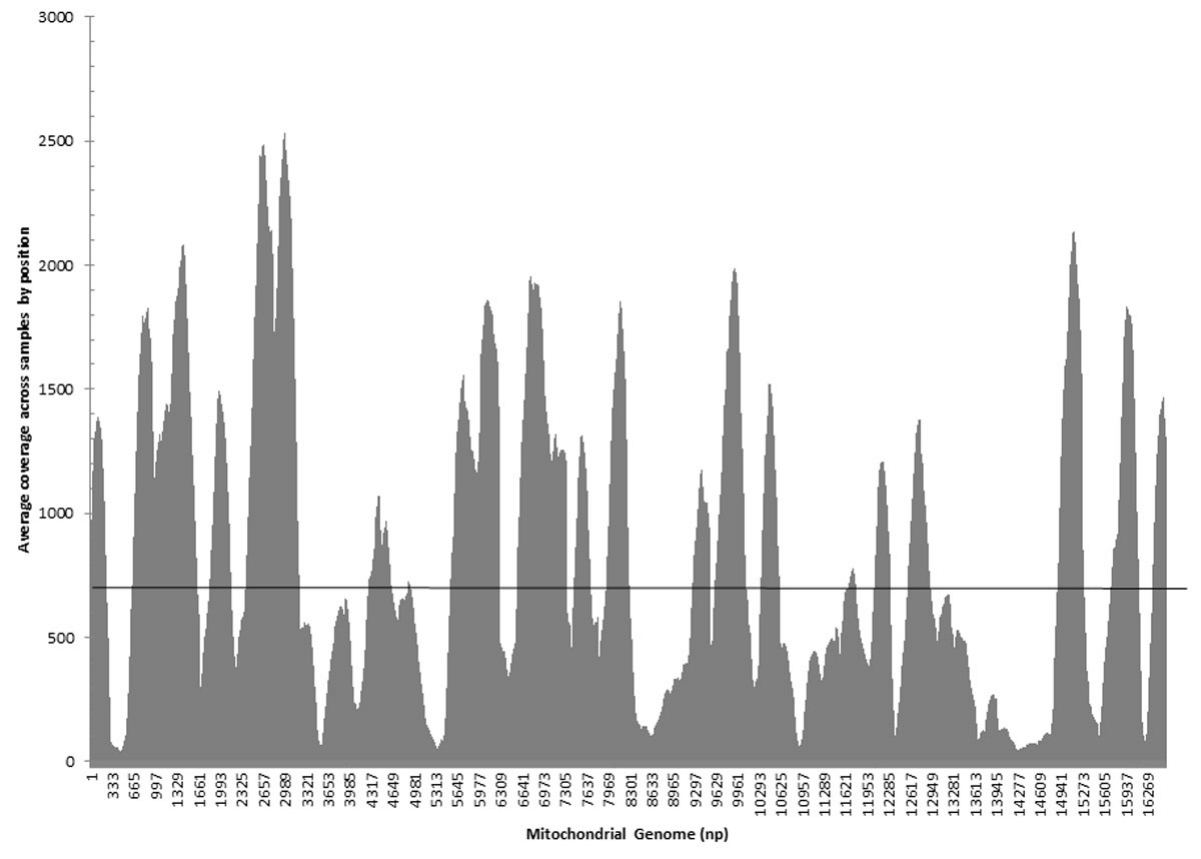
**A concentric Circos plot of the mtGenome.** A concentric Circos plot of the mtGenome representing mean coverage (outer circle; n=24); C homopolymers interrupted by another base (e.g., C_n_TC_n_), per region nucleotide position (middle circle; n=24); and mean coverage differentiated by reverse (dark) or forward (light) strand (inner circle; n=24). The rose diagram in the center is included for nucleotide position orientation and scale bars are included to the left of the individual plots to approximate values. The control region is offset slightly for orientation.

The depth of coverage pattern was similar among all 24 samples. Balanced coverage across nucleotide positions would be ideal. Although the average coverage across samples by position was 810X (± 664), the coverage across the genome varied (Figure 1 and Figure 2). Coverage was consistently low at certain positions and high at other positions across the mtGenome (Additional file 1, Table S1). For example, the coverage of one sample (no. 8) ranged from approximately 25X to 2815X. Given that long PCR was used to generate the amplicons, the variation in coverage cannot be explained as a result of the PCR. The differences in coverage are more likely generated during library preparation and/or sequencing. This range in coverage might be attributed to homopolymeric stretches as these areas may be difficult to sequence due to chemistry-related limitations [20]. Homopolymeric stretches (>3 bases) could be observed along the whole mtGenome (Figure 1). Even though homopolymers were pervasive, accurate sequence results were obtained using the PGM system (excluding potential heteroplasmy). Areas of relatively high (≥810X) and low coverage (≤500X) were investigated further. There were 17 regions with relatively high coverage and 18 regions with low coverage (Additional file 1, Table S1). Areas of low coverage had substantially more C homopolymers (≥2C) than high coverage areas (Table 1). In contrast, the numbers of A, G or T homopolymers were more evenly distributed between high and low coverage regions. Thus, long homopolymers alone did not explain the reduction in coverage. Interestingly, all regions with C homopolymers interrupted by another base (e.g., C_n_TC_n_) displayed relatively low coverage (Figure 2). This motif may impact low coverage, but does not explain all low coverage regions. Further research is necessary to elucidate mechanisms impacting coverage. Regardless, the overall data supported that the PGM system is robust for sequencing mtDNA.

**Table 1:** Comparison of homopolymers between high coverage and low coverage areas across 24 samples.

	High coverage areas^1^	Low coverage areas^2^
Homopolymer type	Number of Homopolymers	Number of Homopolymers

AA	276	320

**CC^3^**	**275**	**347**

GG	167	81

TT	238	225

AAA	104	95

**CCC**	**76**	**124**

GGG	31	12

TTT	82	42

AAAA	22	33

**CCCC**	**31**	**54**

GGGG	4	2

TTTT	18	12

AAAAA	10	11

**CCCCC**	**8**	**28**

GGGGG	2	0

TTTTT	4	3

AAAAAA	4	6

**CCCCCC**	**1**	**8**

GGGGGG	1	0

TTTTTT	2	1

AAAAAAA	2	2

**CCCCCCC**	**0**	**1**

TTTTTTT	1	0

AAAAAAAA	0	1

^1^ Total number of bases in high coverage areas is 6858, and GC content is 44%. ^2^ Total number of bases in low coverage areas is 6760, and GC content is 47%. ^3^ For quick reference, cytosine homopolymers are in bold.

Two methods of fragment shearing during library preparation were tested to determine whether that step of the methodology could affect coverage patterns: physical shearing with the Covaris system and enzymatic shearing with the Ion Shear Plus reagent. Three samples were treated with both methods and the coverage results compared. Overall, the coverage pattern between the shearing methods was similar indicating that coverage variation was likely due to processing subsequent to fragmentation. However, at positions np 621-622 there was a coverage gap (174X to 2365X) in all three samples with physical shearing. However, there was no such gap in coverage in samples treated with enzymatic shearing (513X to 512X). This position is consistent with a primer-binding site. Similar but less extreme drops in coverage also were seen at the other three primer-binding sites. Nakamura et al. [21] suggested that sequence-specific interference favoring dephasing by inhibiting single-base elongation on the Illumina Genome Analyzer II is a factor of sequence coverage variability. Currently, there is no explanation for this one minor difference with PGM generated data, and further study is needed.

### Strand bias

**Figure 3:**
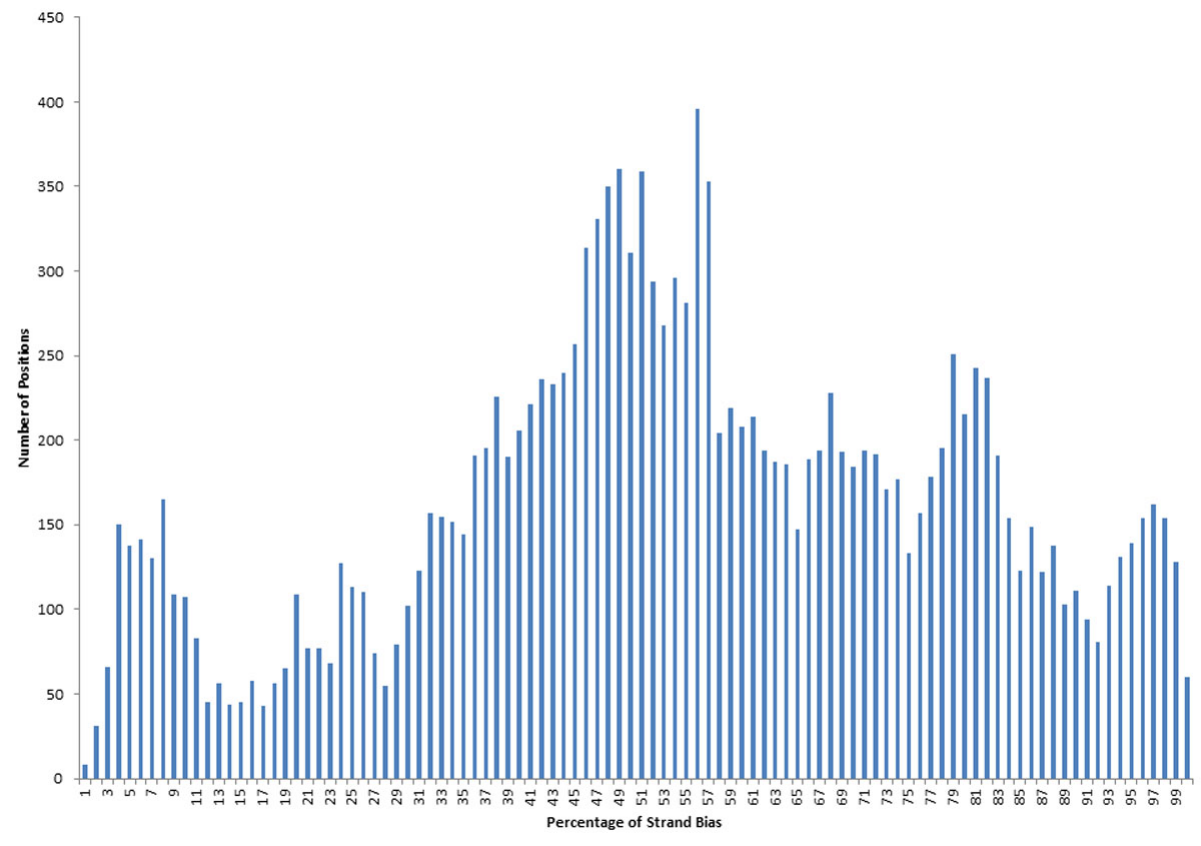
**The overall strand bias display for all 24 samples.** X axis is the ratio of coverage between the forward and reverse strands at each nucleotide position (lower coverage/higher coverage). Y axis is the number of positions with specific percentages of strand bias.

In theory, both strands of a DNA duplex should be sequenced equally. Figure 3 displays the average ratio of coverage between the forward and reverse strands at each nucleotide position (lower coverage/higher coverage). For all 24 samples, two-thirds of the positions of the genome had ratios that were greater than 0.5. A few sites had more extreme strand bias. For example, in one sample (no. 8), out of a total number of 69 reads at np 300, 7 forward direction reads were aligned, while 62 reversed direction reads were aligned; the average strand bias at this position is 0.08. Across the 16,568 nucleotide positions surveyed, 1045 positions showed an average ratio less than or equal 0.1 (Figure 3). While strand bias does not necessarily indicate lower quality data for base calling, balanced strand representation does provide a high degree of confidence that a correct base call was made. In circumstances where in one strand direction there may be an indication of a deletion and in the other strand there is no indication (due to chemistry and/or software), this site would be deemed inconclusive. However, if one strand is over represented, then an incorrect call might arise. Special attention should be given to high strand bias sites and deletions (see below).

### False deletion

**Figure 4:**
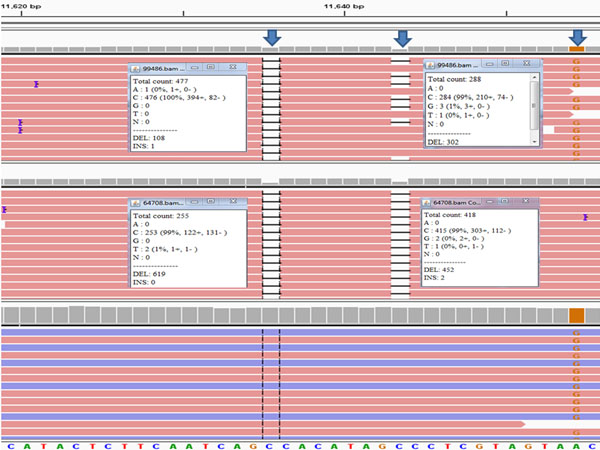
**Deletion pattern at np 11643 in two samples.** Top panel is a PGM result (no. 23); middle panel is a PGM result (no. 22); and bottom panel is a MiSeq result (no. 23). There was no deletion pattern in the MiSeq results.

Parson et al. [17] reported some reads had false deletions in PGM-generated mtDNA sequence data. These deletions could not be verified with Sanger sequencing. However, King et al. [19] did not detect any false deletions with MiSeq data. Therefore, false deletions could be confirmed with concordance testing. In the study herein, we found numerous positions (n=1391) that showed some level of false deletions (Additional file 2, Table S2). These false deletions were measured as a ratio (DR=deletion reads/total reads). In the 16,568 mtDNA nucleotide positions, 156 positions displayed a false deletion of greater than 0.15 in one or more individuals (Additional file 3, Table S3). These false deletions were associated largely with homopolymers (155/156) with a single guanine residue showing a DR of 0.18 in one sample (no. 17). Deletion ratios were observed up to 0.84, although very few positions across the 24 samples had this high of a ratio. The np 11635 had the highest average DR (0.69). In this position, 23 samples showed DR greater than 0.58 except for one sample (no. 23) with only a DR of 0.18. After reviewing the BAM file of this sample in IGV, a variant was observed at the nearby site A11654G (Figure 4). Several positions showed a similar pattern with a variant unique within the dataset that seemed to be associated with a reduction in false deletions. Further study is needed to determine if this SNP variant could somehow be associated with the reduction of DR in this sample.

In some specific regions with 2 consecutive guanine residues (GG), false deletions were observed frequently in PGM sequence results (e.g., nps 6957, 7077 and 12629). In fact, two of the six highest positions in terms of DR and 16/156 positions with high DR (0.15) showed this GG pattern (Additional file 4, Table S4). However, this pattern alone does not account for all false deletions observed. Across the mtGenome, there were 296 GG homopolymers of which only 16 were associated with substantial false deletions. These observations suggested that homopolymers were not the sole cause of this phenomenon, and it likely may be sequence specific. No discernable sequence pattern was observed for these false deletions. The frequency and mechanism of sequence errors has been a subject of other studies. Nakamura et al. [21] showed that sequence specific errors occur in Illumina Genome Analyzer II data, and that these errors were triggered by inverted repeats and GGC motifs. Meacham et al. [22] developed a statistically principled framework and reported that the most common sequence context error is associated with the GGT motif. Furthermore, Allhoff et al. [23] analyzed errors on three different Illumina platforms (GAIIx, MiSeq, HiSeq2000), confirmed previously known error-causing sequence contexts and reported new specific ones. A similar scenario may be occurring with a GG motif described herein for PGM data.

There were no sites where a false deletion represented 100% of the reads. After complete interpretation, none of these sites were assigned as being heteroplasmic. Therefore, correct base calls were obtained. The difference between the two MPS systems could be attributed to chemistry and/or software. False deletions were still present with PGM sequence data that were aligned and called using BWA/GATK. While no incorrect base calls occurred, improvements in chemistry are needed to reduce the phenomenon of false deletions.

### Data accuracy

For the 24 samples analyzed, 31-98 (SNP) variants were observed (each annotated as a difference from the rCRS) per sample. To determine the accuracy of these variant calls, a concordance study was conducted with sequence data generated with the MiSeq system. Of the 24 samples, 23 samples had been sequenced previously on the MiSeq platform [19]. All 1237 (SNP) variants (across the 23 mtGenomes) were concordant between the PGM and MiSeq data, excluding the number of Cs in homopolymers around nps 310 and 16189 regions. These regions are well known sites for heteroplasmic length variants and typically are not used in forensic identifications [24]. Parson et al. [17] reported similar findings in which they described that approximately two-thirds of the different bases (compared with Sanger sequencing data) were observed in or around homopolymeric sequences stretches.

There were three sites worth noting that presented apparent differences between PGM and MiSeq sequence data. One site was the dinucleotide CA insertion at the np 514-524 region. For example, a CACA insertion was predominant in one sample (no. 6) with PGM sequence data; however there were other insertions (CA and CACACA) also present at much lower representation. This region had low coverage and some reads were not sequenced fully. In contrast, data from the MiSeq showed overwhelmingly CACA reads, a relatively small portion of CA (less than observed in the PGM data), and no CACACA reads. Based on this comparison, there is no way currently to indicate whether the minor (CA)_n_ types and their proportions are real, and therefore the lower representation CA variants were considered inconclusive.

**Figure 5:**
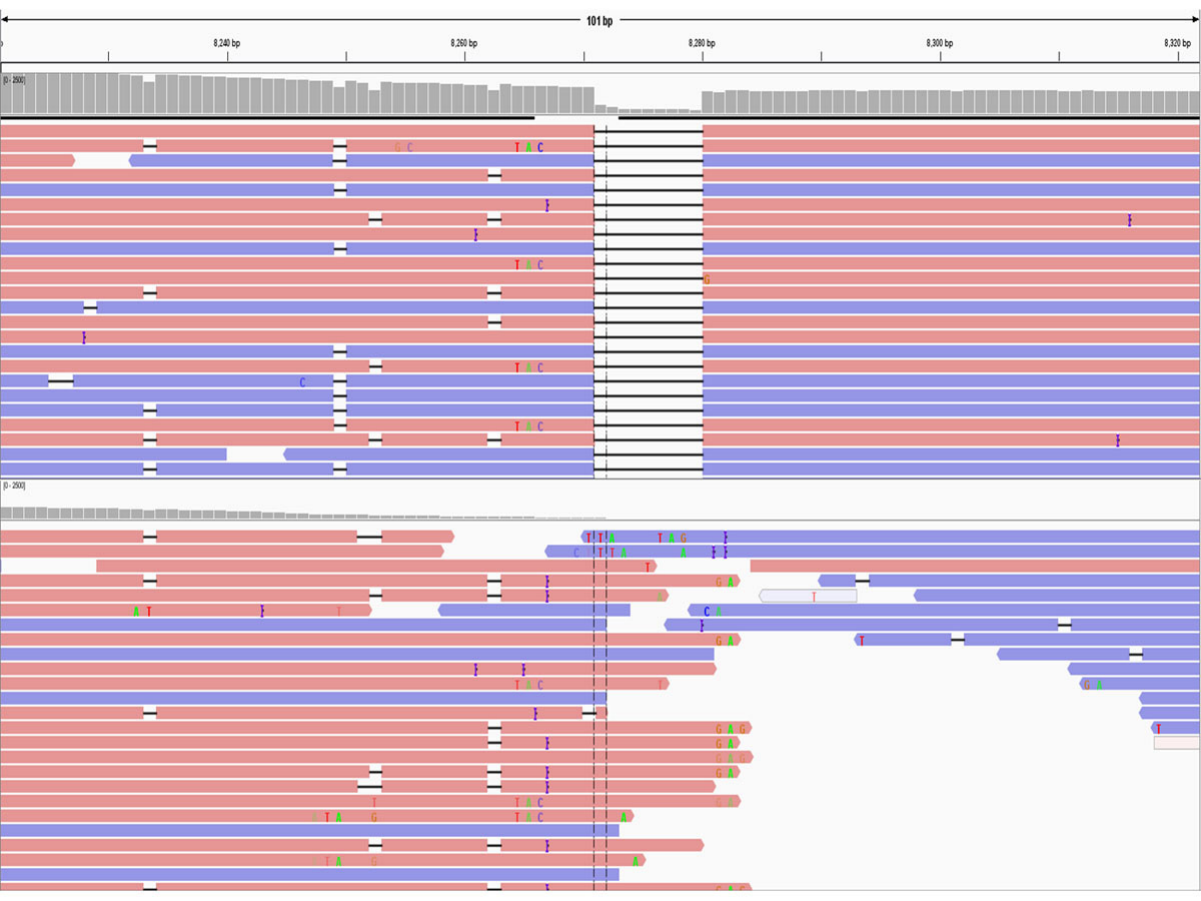
**9-bp deletion pattern was identified in PGM data in one sample (no. 14).** The top panel displays fastq files generated by the PGM aligned using TMAP. The bottom panel displays the same fastq files aligned using BWA.

Another site was a 9-bp deletion of ACCCCCTCT at np 8280-8288 (also known as CCCCCTCTA at np 8281-8289) [25]. The 9-bp deletion was confirmed easily from PGM data (Figure 5). In the PGM workflow, sequence data were aligned with TMAP [26] and variants called using the variant caller v4.0. The MiSeq workflow employed BWA [27][28] to align reads and GATK [29] to call variants. This difference in workflows between the two MPS platforms created a “perceived” difference in insertion/deletion calling because of alignment strategies. The underlying data were the same, but the outputs yielded different nomenclature. To demonstrate this workflow-dependent difference fastq files generated by the PGM were aligned and called using BWA/GATK (Figure 5). Software dependent alignment illustrated the importance of validating bioinformatics workflows in haplotype generation for reliability and consistency among laboratories [17][25]. Lastly, the comparison of sequence data also showed that DR (discussed above) was different between the two platforms, the MiSeq data did not produce any notable false deletion patterns (Figure 4).

### Haplogroup assessment

One way to evaluate accuracy of mtDNA sequencing results was to assess the data in a phylogenetic context. The sequence data were analyzed with HaploGrep software [30] (Additional file 5, Table S5) which provided a ranked list of relevant haplogroups for each sample. Scores >90% supported that the haplogroup assignment was sufficiently reliable. Scores of >80% tended to indicate that the haplogroup assignment was still correct but there may be either sequence information lacking or polymorphism(s) that did not entirely fit the archetypical haplogroup assignment. The scores for all 24 samples ranged between 88.8%-100%. In fact, 23 samples showed scores ≥93.1%. The sample with 88.8% score (no. 12) was assigned to haplogroup L3b1a4 and the variant 1438A->G, which is typically associated with this haplogroup, was not observed in this sample. The result (as well as many other variants across the 24 samples not described herein) was checked and confirmed manually. In addition, the MiSeq sequence result did not display the 1438G variant. Therefore, the SNP state at this site was deemed correct for this sample. A manual review of all sites that do not comport with the haplogroup is a quality control step to achieve high quality mtDNA sequence results. Scores generated by HaploGrep can be used to scrutinize the validity of a variant call, but low scores do not necessarily indicate that the variant call is incorrect. Recurrent mutations do occur and phylogenetic data are based on extant population data [31]. HaploGrep analysis is extremely useful for scouring mtDNA sequence data and with manual reviewing are used to perform a secondary check of results.

## Conclusions

The purpose of this study was to evaluate the performance of PGM for mtGenome sequencing and highlight performance that may need to be addressed for the application of methodology for any discipline that may seek to sequence mtDNA. In this study, mtGenome sequence data generated from the PGM were analyzed and demonstrated to be highly reliable. Depth of coverage variation and strand bias were identified but generally did not impact reliability of variant calls. In addition, multiplexing of samples was demonstrated which can improve throughput and reduce overall cost per sample analyzed. Sequence data generated on the PGM and the MiSeq systems were highly concordant except for the number of Cs in homopolymers around np 310 and 16189 regions, which are not used currently for forensic identifications generated using Sanger methods [24]. More studies are underway to determine regions where sequence data are robust and where they are less reliable (and should be deemed inconclusive); software is being validated; and balance of coverage across the genome is being sought for increased sample multiplexing. The accuracy of mtDNA sequence data was evaluated by analyzing with HaploGrep software. Most samples showed high scores, and those potential sites for further review indicated by the software were evaluated manually to confirm the variant call. Overall, the results of this study supported that whole mtGenome sequence data with high accuracy can be obtained using the PGM platform. The study demonstrated the importance of validation studies to better understand the system(s) used, to highlight potential limitations in specific target regions, and to identify robust and/or inconclusive sequences to refine diagnostic interpretations.

## Methods

### Sample preparation

DNA was extracted from whole blood of 24 volunteers with informed consent. All samples were anonymized to ensure the privacy of the contributing subjects in accordance with University of North Texas Health Science Center IRB. The QIAamp DNA Blood Mini Kit (Qiagen, Hilden, Germany) was used for DNA extraction. The quantity of extracted DNA was estimated using the Qubit® dsDNA BR Assay Kit (Life Technologies) on a Qubit® 2.0 Fluorometer (Life Technologies) following the manufacturer’s recommended protocol [32].

### Library preparation

The entire mtGenome was amplified by long PCR using primers that generate two amplicons approximately 8.5 kb in length in separate reactions as described by Gunnarsdóttir et al. [33]. The PCR included SequalPrep^TM^ 10× Reaction Buffer (Life Technologies), SequalPrep^TM^ 10× Enhancer B (Life Technologies), SequalPrep^TM^ long polymerase (5U/µl) (Life Technologies), DMSO (Life Technologies), primer sets (Life Technologies), DNase-free water, and 5 ng of total genomic DNA according to the manufacturer’s protocol. The amplification conditions were 2 min at 94 °C for polymerase activation, 30 cycles of 10 s at 94 °C for denaturation, 30 s at 60 °C for annealing, 8 min at 68 °C for extension; followed by a final extension of 5 min at 72 °C. The two amplicons were pooled in equimolar amounts (i.e., 50 ng each). The PCR amplicons were enzymatically fragmented using Ion Shear™ Plus Reagents (Life Technologies) and for one experiment by physical shearing with the Covaris system (Covaris, Woburn, MA) following the manufacturer’s protocol. Ion adapters and barcodes were ligated to the fragmented amplicons using the Ion Plus Fragment Library and Ion Xpress™ Barcode Adapters Kits (Life Technologies). The library was size-selected at 315 bp with the Pippin Prep™ instrument (Sage Science, Beverly, MA).

### Template preparation

A diluted library (26 pM) was used to generate template positive Ion Sphere™ Particles (ISPs) containing clonally amplified DNA. Emulsion PCR was conducted using the OneTouch™ 200 Template Kit v2 DL with the Ion OneTouch™ DL configuration (Life Technologies), template-positive ISPs were enriched with the Ion OneTouch™ ES (Life Technologies), and quality of template-positive ISPs was assessed by using the Ion Sphere™ Quality Control Kit (Life Technologies) on the Qubit® 2.0 Fluorometer, following the recommended protocol.

### Sequencing and data analysis

Libraries were sequenced on the Ion 314™ Chip with the Ion PGM™ 200 Sequencing Kit (Life Technologies) following the recommended protocol [34]. Six barcoded samples were sequenced per 314 Chip. All PGM sequences were analyzed with the Ion Torrent Software Suite (v 4.0.2) using the plug-in variant caller (v 4.0). The vcf output of the variant caller was presented in tabular format, as a list of differences to the human mtDNA reference genome, i.e., revised Cambridge Reference Sequence (rCRS). BAM files were visualized with Integrative Genomics Viewer (IGV) [35]. Circos plots were generated using Circos version 0.64 [36]. Whole mtGenome sequence data were compared with mtDNA sequences previously analyzed on the MiSeq [19].

## Abbreviations

STR: short tandem repeat; SNPs: single nucleotide polymorphisms; mtDNA: mitochondrial DNA; mtGenome: mitochondrial DNA genome; MPS: massively parallel sequencing; PGM: Personal Genome Machine; ISPs: Ion Sphere^TM^ Particles; rCRS: revised Cambridge Reference Sequence; IGV: Integrative Genomics Viewer; DR: deletion ratio=deletion reads/total reads.

## Competing interests

The author(s) declare that they have no competing interests.

## Author’s contributions

SBS, XZ, and JLK carried out the PGM sequencing, data analysis, and drafted the manuscript. BB designed this study and revised this manuscript. BLL, MA, MHA and AS contributed in manuscript writing. All authors read and approved the final manuscript.

## Competing Interests

The authors declare that they have no competing interests regarding the subject matter of this article.

## Supplementary Material

Additional file 1 **Table S1** The positions with relatively high coverage (≥810X) and position with low coverage (≤500X). Click here for file

Additional file 2 **Table S2** Positions with false deletion ratio greater than or equal to 0.01. Click here for file

Additional file 3 **Table S3** 156 positions showing high false deletion ratio. Click here for file

Additional file 4 **Table S4** Homopolymer types showing high false deletion ratio. Click here for file

Additional file 5 **Table S5** Haplogroup assignment based on HaploGrep software analysis. Click here for file

## Declarations

This work was supported in part by award no. 2012-DNBXK033, awarded by the National Institute of Justice, Office of Justice Programs, U.S. Department of Justice. The opinions, findings, and conclusions or recommendations expressed in this publication are those of the authors and do not necessarily reflect those of the U.S. Department of Justice. The authors also would like to thank Life Technologies for the support of reagents for this study. AS would like to thank Finland’s Foundations’ Professor Pool and Paulo Foundation for support. The Centre of Excellence in Genomic Medicine Research, King AbdulAziz University, Jeddah, Saudi Arabia, paid the publication costs for this article.

This article has been published as part of *BMC Genomics* Volume 16 Supplement 1, 2015: Selected articles from the 2nd International Genomic Medical Conference (IGMC 2013): Genomics. The full contents of the supplement are available online at http://www.biomedcentral.com/bmcgenomics/supplements/16/S1
